# A Comprehensive Review of the Importance of the Main Comorbidities in Developing Cognitive Disorders in Patients With Spinal Cord Injuries

**DOI:** 10.7759/cureus.70071

**Published:** 2024-09-24

**Authors:** Nefeli Anna Papageorgiou, Platon Papageorgiou, Aikaterini Kotroni, Elias Vasiliadis

**Affiliations:** 1 Physical Medicine and Rehabilitation, KAT General Hospital, Athens, GRC; 2 Orthopedics and Traumatology, University of Patras, Patras, GRC; 3 Physical Medicine and Rehabilitation, KAT Hospital, Athens, GRC; 4 3rd Department of Orthopaedic Surgery, KAT Hospital, National and Kapodistrian University of Athens School of Medicine, Athens, GRC

**Keywords:** cognitive impairment, rehabilitation, spinal cord comorbidities, spinal cord injury, traumatic spinal cord injury

## Abstract

Spinal cord injuries (SCI) refer to lesions in the spinal cord due to direct trauma (traumatic SCI-TSCI), such as vehicle accidents, falls, and violent events, or other pathological conditions (non-traumatic SCI) such as metabolic disorders, inflammation, or degenerative disorders. Depending on the location of the injury, patients may experience movement and/or sense disabilities in their lower limbs, torso, or upper limbs. Even though poorly studied, it has been found that such patients have a higher risk of developing cognitive disorders, such as deficits in concentration, short and long memory, comprehension, and problem-solving, as well as mental deficits in the form of difficulty socializing and expressing emotions. The main contributing factor of cognitive impairment in patients with SCI has been identified as a traumatic brain injury (TBI), but other comorbidities play an important role. In spite of the correlation that has been found between certain comorbidities and cognitive impairment in patients with SCI, further investigation into the importance of these pathological states as well as future research into the approach of these patients is necessary.

## Introduction and background

According to the World Healthcare Organization (WHO), it is estimated that 250,000-500,000 new cases of spinal cord injury (SCI) arise per year. Spinal cord injuries may cause many systemic complications, affecting multiple organs [1]. Spinal cord injuries are assessed using the American Spinal Injury Association (ASIA) impairment scale to identify the anatomical dysfunction and the functional independence measure (FIM) to quantify the functional deficit of the patient [2,3]. Adults with SCI were nearly 13 times more likely to have cognitive impairment than those without making cognitive deficit a significant reason to be concerned [4]. Most SCI cases occur after mechanical causes such as car accidents, falls, and violent incidents. Spinal cord injury beyond the spinal cord affects other systems of the human body by disrupting their function. Chronic pain, observed in up to 94% of patients, and chronic fatigue are also described [5]. Another important and life-threatening condition among patients with a level of SCI above the T6 is autonomic dysreflexia (AD) [6]. Cell death and tissue injury begin immediately after the mechanical event, preceded by an inflammatory process, leading to the destruction of the nerve tissue [7]. Mental functions are a higher-level domain of brain function. The types of mental functions are attention, memory, visuospatial functions, language and verbal skills, executive functioning, etc. [8]. The main cause of mental impairment is damage to neural tissue; a cognitive deficit may have a short-term or progressive nature, depending on the leading cause [9]. Patients with SCI have a 13 times higher risk of developing cognitive decline [10]. The purpose of this review is to present the current knowledge that we have acquired in the field of cognitive disorders in patients with SCI, specifying the main comorbidities these patients tend to present.

## Review

Materials and methods

A literature search was performed using the PubMed database (June 29, 2024) with the following search phrase: (spinal cord injury or paraplegia or tetraplegia) and (cognitive impairment or cognitive function or comorbidities). The authors screened the titles and abstracts independently, ensuring that the inclusion criteria were met before proceeding to the full text. A manual search was also performed within the references of important articles. The areas of interest were the comorbidities following spinal cord injuries and their role in cognitive impairment. The types of articles that were excluded were systematic reviews, literature reviews, meta-analyses, and case reports. The exclusion criteria were non-cognitive impairment-related comorbidities in spinal cord injuries, animal trials, cadaveric studies, non-English language, surveys, and non-empirical studies (e.g., opinion articles).

Results

**Figure 1:**
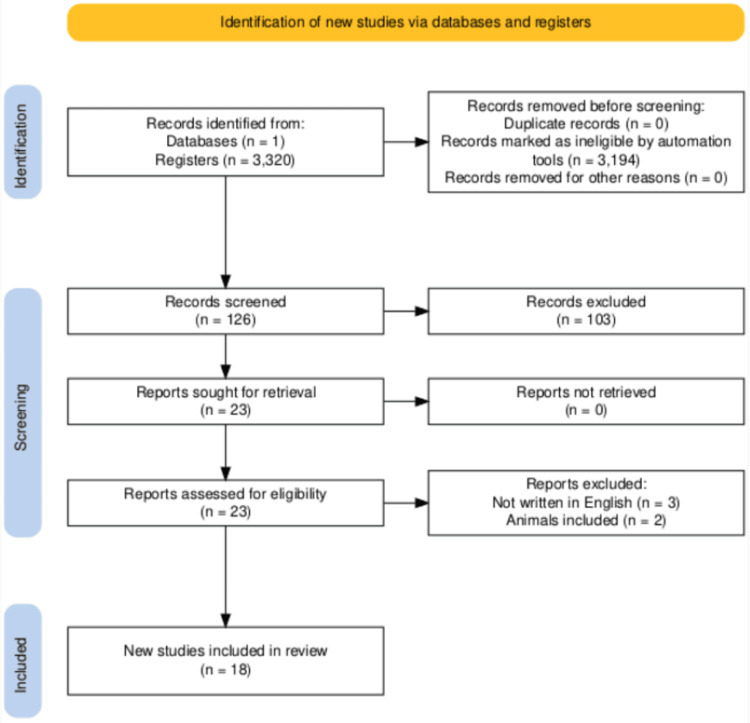
PRISMA flow chart: the main results of studies according to the studied comorbidities The study followed the PRISMA standards of 2022. PRISMA: Preferred Reporting Items for Systematic Reviews and Meta-Analyses

After adding the criteria mentioned above, the search provided a total of 126 studies. Ranging from 1955 to 2024 from which 108 were excluded after reading the abstracts. Studies focusing on other than the comorbidities of spinal cord injuries in patients with cognitive disorders, studies being conducted in another language, and studies including animals were eliminated. In total, 18 scientific documents were included in the review (Table 1). The flowchart summarizing the above search procedure is presented in Figure 1.

**Table 1:** Main results of studies according to studied comorbidities

Studies	Comorbidities	Symptoms
Macciochi et al. 2012 [11], Tolonen et al. 2007 [12], Mollayeva et al. 2020 [13], Heled et al. 2022 [14]	Traumatic brain injury	Severity of traumatic brain injury
Phillips et al. 2014 [15], Jegede et al. 2010 [16], Wecth et al. 2020 [17], Handrakis et al. 2015 [18]	Autonomic nervous system	Cerebral blood flow, hypotension, thermoregulation
Craig et al. 2015 [19], Lim et al. 2017 [20], Wan et al. 2020 [21], Pozzato et al. 2023 [22]	Mental disorders	Depression and anxiety, traumatic spinal cord injury
Krebs et al. 2018 [23]	Neurogenic bladder drugs	Antimuscarinic drugs: solifenacin, darifenacin
Murray et al 2007 [24]	Pain	Chronic pain
Sajkov et al. 1998 [25], Schembri et al. 2017 [26]	Sleep-disordered breathing	Night desaturation, severity of obstructive sleep apnea
Mahmoudi et al. 2021 [27]	Age	Alzheimer disease
Allison et al. 2016 [28]	Chronic inflammation	Proinflammatory mediators

Discussion

Traumatic Brain Injury (TBI)

In 2012, the severity of TBI was also studied by Macciochi et al., showing that only severe or moderate TBI may provoke cognitive impairment in patients with SCI when tested with the FIM scale [11]. In a cross-sectional study by Tolonen et al. in 2007, about 67% of patients with SCI and concomitant TBI showed cognitive impairment when tested with a neuropsychological test battery [12]. Furthermore, the same study underlines that TBI is underdiagnosed in patients with SCI. [12].

Mollayeva et al., in 2020, after testing two groups (TBI and TBI with SCI) with a cognitive subscale of the FIM score, concluded that patients with concomitant TBI and SCI did not ameliorate in terms of problem-solving and comprehension, notifying that SCI alone may have a negative impact on functioning outcomes in rehabilitation program [13].

A recent study by Heled et al. mentions the importance of TBI screening among patients with SCI and distinguishing traumatic SCI (TSCI) from non-traumatic SCI in those patients to clarify TBI’s relationship with the appearance of cognitive dysfunction in those patients [14]. Furthermore, when comparing a group of 22 individuals with chronic traumatic SCI and a control group (non-SCI), they found that the first group had lower scores in all executive functions apart from naming, underlying the importance of cognitive evaluation in SCI patients regardless of the incidence of TBI [14].

Autonomic System

The study of Phillips et al. implies that altered cerebral blood flow (CBF), illustrated by cerebral hypoperfusion along with impeded neurovascular coupling and cerebral blood flow regulation, may also affect cognitive function and may be improved by normalizing average blood pressure [15]. It is mentioned that midodrine, an alpha-adrenergic agonist, has a beneficial effect on cognitive function in a group sample of 10 patients with SCI [15]. Blood pressure dysregulation and the resulting protracted hypotensive condition, which can last for more than 24 hours, may play a part in the development of the cognitive impairment seen in individuals with spinal cord injuries by lowering cerebral blood flow to specific brain regions [16]. The study by Jegede et al. (2010) showed that people with SCI who had low blood pressure, even without experiencing any symptoms, performed worse on memory and attention tasks as compared to those who had normal blood pressure [16]. When in 2020, Wecht et al. investigated the effect of midodrine, an alpha-adrenergic agonist, in CBF during cognitive tests in patients with SCI, they concluded that the increase of systolic blood pressure and in CBF velocities may have a positive impact on cognitive function along time [17]. Another domain affected due to autonomic system dysfunction is thermoregulation, for which Handrakis et al. tested cognitive function in seven patients with tetraplegia while exposed to cold temperatures (18° Celsius). In comparison to the control group (uninjured individuals), the SCI group scored lower in cold temperatures, concluding that when thermoregulation is disturbed, this may be a factor of cognitive dysfunction [18].

Mental Disorders

Whether people with psychiatric disorders are more prone to SCI or whether SCI predisposes people to develop a psychiatric illness remains controversial, and more research is required. Craig and his colleagues reported in 2015 that 17%-25% of SCI patients were diagnosed with a psychiatric condition at the beginning of the rehabilitation program while this percentage did not change significantly upon discharge from the rehabilitation clinic [19]. Among psychiatric diseases, depression was the most common psychiatric disorder, followed by bipolar disorder and generalized anxiety disorder, which occurs in 4.2%, and post-traumatic stress disorder (PTSD), which occurs in 1.4% of patients, when measured six months post-discharge. Alcohol abuse was present in about 8.6% of patients [19]. Interestingly, TSCI increases the risk of the development of psychiatric disorders. First, Lim et al., in 2017, mentioned the high risk of depression and anxiety appearance in patients with TSCI after their discharge [20]. Three years later, Wan et al. (2020), when comparing patients with TSCI, found that 33% of them developed a mental disorder, whereas among SCI patients from a non-traumatic reason, this percentage was only 3%. The most notable disorders were depression, anxiety, and bipolar disorder; it is mentioned that rehabilitation interventions may have a beneficial role [21]. Another recent study by Pozzato et al. shows that self-reported cognitive impairment by patients with SCI (n=41) when using the Cognitive Failures Questionnaire, was strongly related to their comorbidities, the strongest of which was depressive mood [22].

Antimuscarinic Drugs

Many patients with SCI take anticholinergic medication for bladder dysfunction, especially for neurogenic detrusor overactivity. Krebs et al. assessed the possible effect on cognitive function in individuals with SCI, who were taking antimuscarinic drugs. The neuropsychological test between 19 SCI patients not taking antimuscarinic treatment (= control group) and 10 SCI treated with antimuscarinic drugs was not different. The only domain affected in the second group was immediate recall, and both groups had better scores at the follow-up assessment than in the primary one [23]. Even though Krebs et al. did not find a negative effect of antimuscarinic drugs, this underlies the importance of further studies, as it is well-known that oxybutynin, another antimuscarinic drug, negatively impacts cognitive function in the elderly.

Pain

When Murray and colleagues tested the perception before and after SCI of cognitive, physical, and quality of life (QOL) in 63 patients, they found a noticeable change [24]. Additionally, it is mentioned that pain has a negative impact on functioning, whereas SCI also decreases the feeling of happiness in the long term among patients with SCI [24].

Sleep-Disordered Breathing

Sajkov et al. studied the impact of sleep-disordered breathing in the SCI population with a sample of 37 SCI patients. They found that 19% of patients with tetraplegia had night desaturation (O_2 _<80%) and reported to present with impaired attention, concentration, impaired immediate and short-term memory, mental flexibility, and working memory. The most common type of sleep-disordered breathing was obstructive sleep apnea [25]. According to Schembri, there is a direct correlation between sleep apnea severity and cognitive decline. More recently, Schembri et al. classified participants with tetraplegia (with no TBI) based on the severity of sleep apnea and administered a similar battery of tests. Participants with more severe sleep apnea performed worse on the attention, information processing, and immediate recall tasks compared to mild/ moderate groups. [26]

Age

A crucial study by Mahmoudi et al. (2021) showed that a spinal cord injury could double the incidence of Alzheimer's disease in people aged 45-64 years and increase it in older subjects (>65 years old) with SCI when compared to healthy subjects [27].

Chronic Inflammation

Inflammation occurring after SCI may be another contributing factor to explain cognitive decline in patients with SCI. The clinical trial of Allison et al. included adult patients with various types and severity of chronic SCI were included in this study. After a 12-week trial, including an anti-inflammatory diet, and tested by the California Verbal Learning Test (CVLT), a decrease of proinflammatory mediators was seen but not a significant change in CVLT. The same study underlies the importance of the volume of the hippocampus to evaluate verbal learning and memory [28].

Other factors, such as gender, social, and demographic variables, educational level, and the history of learning difficulty before the injury, are important factors in the appearance and development of mental impairment after SCI.

Overall, adults with SCI are frequently at higher risk for cognitive impairment than those without SCI. Cognitive deficits are present in the subacute stage and worsen with time. From a clinical point of view, we confirmed that comorbidities may be a contributing factor to apparent cognitive impairment. More specifically, TBI was found to be the most significant contributor to cognitive disorders in patients with SCI, and its diagnosis is crucial. The dysregulation of the autonomic nervous system is another important factor, as decreased CBF and abnormal thermoregulation may be correlated with cognitive deficits. Among psychiatric disorders, depression and anxiety seem to have a strong relationship with cognitive decline, whereas the assurance of mental health is undeniably of primary importance to patients with SCI. According to the literature, the extent to which antimuscarinic drugs affect cognitive function is still under debate. As pain is a frequent comorbidity, it may have a negative impact on cognition in patients with SCI. Patients with sleep-disordered breathing are more susceptible to cognitive decline while advanced age predisposes to the onset of mental decline and could be the main risk factor for its onset. In conclusion, chronic inflammation after injury is still under research to clarify its role in cognitive function.

## Conclusions

The study results emphasize the extreme importance of identifying the comorbidities of SCI that may further lead to cognitive impairment. While several mechanisms have been proposed to explain cognitive dysfunction in individuals with SCI, no single explanation has been pinpointed; instead, a combination of factors is likely responsible. Further research is required to identify and clarify the major causative factors of cognitive impairment in SCI.
